# A Longitudinal Electromyography Study of Complex Movements in Poststroke Therapy. 2: Changes in Coordinated Muscle Activation

**DOI:** 10.3389/fneur.2017.00277

**Published:** 2017-07-20

**Authors:** Negin Hesam-Shariati, Terry Trinh, Angelica G. Thompson-Butel, Christine T. Shiner, Penelope A. McNulty

**Affiliations:** ^1^Neuroscience Research Australia, Sydney, NSW, Australia; ^2^School of Medical Science, University of New South Wales, Sydney, NSW, Australia

**Keywords:** muscle synergy, non-negative matrix factorization, upper limb, rehabilitation, chronic stroke

## Abstract

Fine motor control is achieved through the coordinated activation of groups of muscles, or “muscle synergies.” Muscle synergies change after stroke as a consequence of the motor deficit. We investigated the pattern and longitudinal changes in upper limb muscle synergies *during* therapy in a largely unconstrained movement in patients with a broad spectrum of poststroke residual voluntary motor capacity. Electromyography (EMG) was recorded using wireless telemetry from 6 muscles acting on the more-affected upper body in 24 stroke patients at early and late therapy during formal Wii-based Movement Therapy (WMT) sessions, and in a subset of 13 patients at 6-month follow-up. Patients were classified with low, moderate, or high motor-function. The Wii-baseball swing was analyzed using a non-negative matrix factorization (NMF) algorithm to extract muscle synergies from EMG recordings based on the temporal activation of each synergy and the contribution of each muscle to a synergy. Motor-function was clinically assessed immediately pre- and post-therapy and at 6-month follow-up using the Wolf Motor Function Test, upper limb motor Fugl-Meyer Assessment, and Motor Activity Log Quality of Movement scale. Clinical assessments and game performance demonstrated improved motor-function for all patients at post-therapy (*p* < 0.01), and these improvements were sustained at 6-month follow-up (*p* > 0.05). NMF analysis revealed fewer muscle synergies (mean ± SE) for patients with low motor-function (3.38 ± 0.2) than those with high motor-function (4.00 ± 0.3) at early therapy (*p* = 0.036) with an association trend between the number of synergies and the level of motor-function. By late therapy, there was no significant change between groups, although there was a pattern of increase for those with low motor-function over time. The *variability accounted for* demonstrated differences with motor-function level (*p* < 0.05) but not time. Cluster analysis of the pooled synergies highlighted the therapy-induced change in muscle activation. Muscle synergies could be identified for all patients during therapy activities. These results show less complexity and more co-activation in the muscle activation for patients with low motor-function as a higher number of muscle synergies reflects greater movement complexity and task-related phasic muscle activation. The increased number of synergies and changes within synergies by late-therapy suggests improved motor control and movement quality with more distinct phases of movement.

## Introduction

Fine motor control of the upper limb requires complex movements based on multiple degrees of freedom that permit movement variability and versatility (1, 2). The central nervous system controls such complex motor tasks by coordinated activation of groups of muscles, referred to as “muscle synergies” (3–6). The combination of the brain and spinal circuitry is essential for the simultaneous recruitment of multiple muscle synergies that explain a wide range of movement patterns (7, 8). Muscle synergies have been extracted from electromyography (EMG) recordings to define movements in both animals including frogs (9, 10), rats (11), cats (12–14), and monkeys (15); and humans with reference to gait (16–18), balance and posture (19, 20), hand function and posture (21, 22), arm movements (2, 7, 23), and isometric force (24, 25).

Multiple temporal synergy profiles are weighted and integrated to define coordinated muscle activation during a task (2). Muscle synergies can include any number of muscles and each muscle can contribute to multiple synergies (8). Muscle synergies have been investigated in acute, subacute (26–28), and chronic stroke (17, 23, 29) showing abnormalities compared to healthy people (18, 30, 31). Such changes reflect poststroke motor impairment which can be attributed in large part to disorders in the neural pathway (8), reduced corticospinal drive (32), disuse atrophy (33), and loss of independent joint control and impaired motor coordination (29).

Muscle synergy analysis has detected poststroke abnormalities in the number, structure, and recruitment profile of muscle synergies. For example, the number of muscle synergies recruited in the poststroke gait cycle was reduced in patients with more severe impairment and in comparison to healthy subjects (17, 34). This presumably reflects a change in the number of independent motor subtasks given the standard analysis of the gait cycle in four distinct phases and the use of four synergies for healthy subjects and patients with high motor-function.

Several analysis algorithms have been suggested for the decomposition of muscle activation profiles into muscle synergies. Tresch and colleagues (35) evaluated and compared different matrix factorization methods including factor analysis, independent component analysis alone and applied to principle component analysis, and non-negative matrix factorization (NMF). The authors concluded that these methods identify muscle synergies very similar to one another. In this study, we implemented NMF which has commonly been used to detect muscle synergies from EMG activation (17, 25, 36, 37). NMF quantifies muscle synergies as a linear combination of the timing profile and a weighting assigned to each muscle involved in each synergy.

Few studies have examined the changes in poststroke muscle synergies with rehabilitation, but see Ref. (28). In this study, we extracted muscle synergies during a complex task to investigate the changes in muscle activation profiles (i.e., muscle synergies) in chronic stroke during an intensive 14-day protocol, in this case Wii-based Movement Therapy (WMT) (38, 39). This therapy is as effective as the current best practice in stroke rehabilitation, Constraint-induced Movement Therapy (38, 40). The primary aim of this study was not the therapy itself, but to quantify poststroke muscle synergies during therapy. Muscle synergy analysis cannot be used to investigate recovery mechanisms occurring in the brain but was used here as a neurophysiological indication to distinguish the level of impairment and the effect of therapy on coordinated muscle activation (41). To identify some of the neuromuscular mechanisms underpinning the improvement reported using clinical motor-function assessments (38, 39), NMF was applied to the EMG recorded from six muscles of the more-affected arm and upper body during the Wii-baseball component of WMT. This longitudinal study examined EMG at early and late therapy, and at 6-month follow-up for a subset of patients. We hypothesized that the number of muscle synergies would be correlated with the level of motor-function after stroke and that the number of synergies would change with therapy.

## Materials and Methods

### Participants

**Figure 1:**
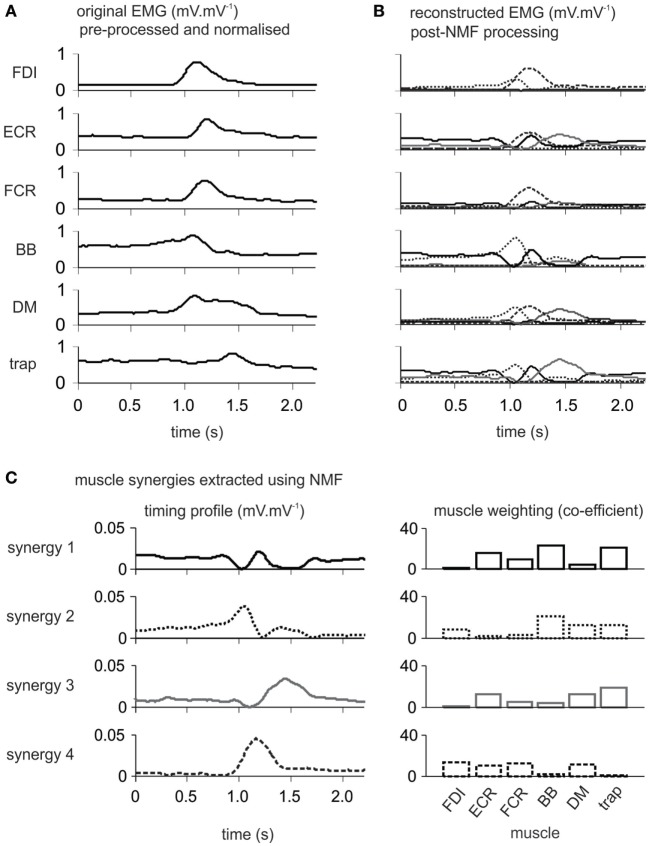
Single patient electromyography (EMG) data showing the progression through analysis using non-negative matrix factorization (NMF). The 61-year-old male patient with moderate motor-function was 5 months poststroke. **(A)** Preprocessed normalized EMG from six upper body muscles on the more-affected side during Wii-baseball swings prior to processing by the NMF algorithm. **(B)** Reconstructed EMG after processing by NMF as the integration of muscle synergies for each muscle. **(C)** Each derived muscle synergy is a combination of the timing profile and muscle weightings. FDI, first dorsal interosseous; ECR, extensor carpi radialis; FCR, flexor carpi radialis; BB, biceps brachii; DM, deltoid medius; trap, trapezius (middle portion).

Twenty four patients (16 males, 8 females) aged 37–80 years (57.9 ± 12.1, mean ± SD) and 3–88 months poststroke (26.7 ± 4.3, mean ± SE) were randomly selected from a larger cohort who were consecutively recruited from St. Vincent’s and Prince of Wales’ Hospitals, Sydney [the same patients as presented in Hesam-Shariati et al. (42), see Table 1 for a summary of baseline characteristics]. All participants were hemiparetic following either an ischemic or hemorrhagic stroke and were classified into three groups of low, moderate, or high motor-function based on their ability to perform two tests of upper limb manual dexterity (43). The inclusion criteria were as before: ≥10° of voluntary movement in at least one digit of the more-affected hand; cognitive competency measured as a Mini-Mental State Examination score ≥24; suitable skin for sensor placement; and the ability to communicate in English. Exclusion criteria included unstable blood pressure; comorbidities affecting upper limb sensorimotor function; and engagement in any other formal upper limb rehabilitation program. All participants gave signed informed consent to the study that was approved by the St. Vincent’s Hospital Human Research Ethics Committee, Sydney, and conducted in accordance with the Declaration of Helsinki. Ten of the 24 patients could not attend the 6-month follow-up session for a range of reasons including: return to work, too far to travel, and unrelated health problems. The data for one patient were excluded for technical issues [see Ref. (42), Figure 1]. As detailed in Hesam-Shariati et al. (42), the clinical assessment results for all patients (but not NMF analyses) have been published previously [see Ref. (44) *n* = 20; (38) *n* = 9; and (40) *n* = 8].

### Therapy

WMT is a standardized 14-day program focused on the more-affected upper limb, which consists of 1-h of formal therapy on 10 consecutive weekdays delivered by an Accredited Exercise Physiologist, augmented by prescribed home practice starting on day 2 and increasing throughout the program [see Figure 2A in Ref. (42)]. This therapy uses the Nintendo Wii and Wii-Sports games (Nintendo, Japan) as a rehabilitation tool that targets movement quality and independence in activities of daily living (38, 39). The movements required in Wii-Sports were modified according to the capacity, range of motion, and strength of each patient. Although WMT games include Wii-golf, -baseball, -tennis, -bowling, and -boxing, the analyses of this study were applied only to Wii-baseball swings. Each patient played two or three games of Wii-baseball during each session of therapy. This Wii-baseball movement was selected for analysis for several reasons. First, all patients were able to complete this game, regardless of the level of residual voluntary motor-function. Second, the game determines the onset of each movement by pitching the ball. This allowed individual movements to be identified more clearly in the EMG signal. Finally, the nature of the game provided the most consistent task demands.

### EMG Recording

Surface EMG was recorded from six muscles of the upper body on the more-affected side: trapezius (middle portion), deltoid medius, biceps brachii (BB), extensor carpi radialis, flexor carpi radialis, and first dorsal interosseus (FDI) using Trigno wireless sensors (Delsys, USA). The data were collected continuously during formal WMT sessions at early (day 2–3) and late (between days 12–14) therapy, and in a subset of patients, again during the 6-month follow-up session. Each EMG sensor contains four silver bar electrodes, arranged in two pairs with an interelectrode pair distance of 10 mm. The sensor is designed to maximize the detection of muscle activation in a field perpendicular to the muscle fibers. Data were amplified 300 times, filtered between 20 and 450 Hz, and sampled at 2 kHz using EMGworks (Delsys, USA) as per intrinsic device settings.

### Clinical Motor-Function Assessments and Game Performance

The efficacy of WMT was evaluated using clinical motor-function tests as described in Hesam-Shariati et al. (42) including the Wolf Motor Function Test (WMFT) (45), upper limb motor Fugl-Meyer Assessment (FMA) (46), and the Motor Activity Log Quality of Movement scale (MALQOM) (47). The Wii-baseball game performance was assessed as the number of hits, regardless of the outcome according to the rules of baseball, and this was recorded during therapy. However, the primary goal of therapy was movement quality and not game performance. The average duration of each swing for each trial was measured in seconds.

### Data Analysis

#### EMG Preprocessing

Electromyography signals were DC removed, root mean square processed using a sliding 50 ms window and demeaned using Spike2 software (CED, UK). Mean baseline EMG was measured over 1 s prior to the beginning of the Wii-baseball game, while the muscles were at rest. The mean was subtracted from the signal of the same game for each patient. To enable comparison between patients, the EMG of each muscle was normalized to its peak amplitude, then averaged over 10 consecutive Wii-baseball swings for each patient at early and late therapy and at 6-month follow-up.

#### Non-Negative Matrix Factorization

Muscle synergies were extracted from the EMG signals using the NMF method (4, 35, 48, 49). This optimization method was applied to the EMG recordings of the six muscles using the in-built nnmf function in MATLAB R2014a (MathWorks, USA). Random initial values were generated as the input for the multiplicative algorithm of the function, the output of which provided the initial values of the alternating least squares (ALS) algorithm (50). Then, the ALS algorithm was used to characterize the EMG of the six recorded muscles (*m* = 6) as a lower-rank combination of the relative weighting (*W*) of each muscle and the timing profile (*H*) of each synergy (equation below) in a complex movement (see Figure 1).

$$\text{EMG}≅\sum_{k=1}^{m-1}W_{k}H_{k}(t)$$

#### Number of Muscle Synergies

The number of muscle synergies needed to define coordinated muscle activation in a complex movement was determined using the term *variability accounted for* (VAF) (14, 17) and the mean squared error (MSE) term (9, 51) according to the formula below. The VAF is defined as 100 times the squared correlation coefficient between the original EMG (EMG_o_) and the reconstructed EMG (EMG_r_) from the NMF algorithm (23). The minimum number of synergies was identified when VAF increased by less than 2% when another synergy was added. VAF for the acceptable number of synergies was required to be greater than 97% while the MSE was less than 10 × 10^−4^.

$$\text{VAF}=100\times \left(1-\frac{\sum {\left({\text{EMG}}_{\text{o}}-{\text{EMG}}_{\text{r}}\right)}^{2}}{\sum {\left({\text{EMG}}_{\text{o}}-\bar{{\text{EMG}}_{\text{o}}}\right)}^{2}}\right)$$

$$\text{MSE}=\frac{1}{n}\sum_{1}^{n}{\left({\text{EMG}}_{\text{o}}-{\text{EMG}}_{\text{r}}\right)}^{2}$$

#### Similarity of Synergy Timing Profiles

The similarity between individual synergy timing profiles from each subject on a group basis was quantified using the scalar product (1, 31). More than 50% of patients used four distinct synergies to account for the variability of muscle activation at early and late therapy and at 6-month follow-up. The analysis of similarity between timing profiles requires the same number of synergies from each patient to be entered in the analysis to enable the comparison of synergy profiles. Thus, regardless of the actual number of synergies, four synergies were extracted from the muscle activation of all patients. Then, one set of four synergies from one subject was randomly selected in each motor-function group and used as the template. The synergy timing profiles from all other subjects within the same motor-function group at each time point were matched to provide the highest scalar product between two synergies. The scalar product (*r*-value) is a measure of the similarity in which one numerical vector is projected onto another, so that an *r*-value of 1 represents complete similarity and a value of 0 represents the absence of any similarity.

#### Clustering Synergy Structures

The muscle weightings of the actual synergies from all subjects were pooled to be categorized using cluster analysis (23, 51) at early and late therapy. This procedure was performed using the in-built functions from the MATLAB statistics toolbox. Euclidean distance was used to measure the similarity between pairs of muscle weightings. The minimum number of clusters was determined based on grouping synergies when there was no more than one synergy from a subject in each cluster. Cluster analysis requires the inclusion of all extracted synergies to avoid overlap and to merge the analysis to a limited and realistic number of clusters. This method avoids the inclusion of more than one synergy from each patient in each cluster.

### Statistical Analysis

A potential relationship between the number of muscle synergies and the level of motor-function was investigated using Pearson chi-square test, Fisher’s exact test, and linear-by-linear association. If more than 20% of the cells had an expected count <5, the *p*-value of Fisher’s exact test was reported instead of Pearson chi-square. In addition, linear-by-linear association was used to reveal trends in larger than 2 × 2 tables. The same tests were used after the cluster analysis to evaluate the incidence of each cluster in each level of motor-function.

A mixed-effect model was implemented for any given number of synergies (range 1–5) to detect the effect of motor-function level (low, moderate, high) and time (early therapy, late therapy, follow-up) on VAF. This model is powerful and flexible with missing data (i.e., to account for *n* = 13 at follow-up). Clinical assessments and game performance data were analyzed using paired *t*-test (for parametric data) and Wilcoxon signed-rank test (for non-parametric data) to compare means between time points. Statistical analyses were conducted in SPSS 23 software (IBM, USA) and the differences were considered significant when *p* < 0.05.

## Results

### Number of Synergies Extracted from Wii-baseball

#### Difference in the Number of Synergies across Groups

**Figure 2:**
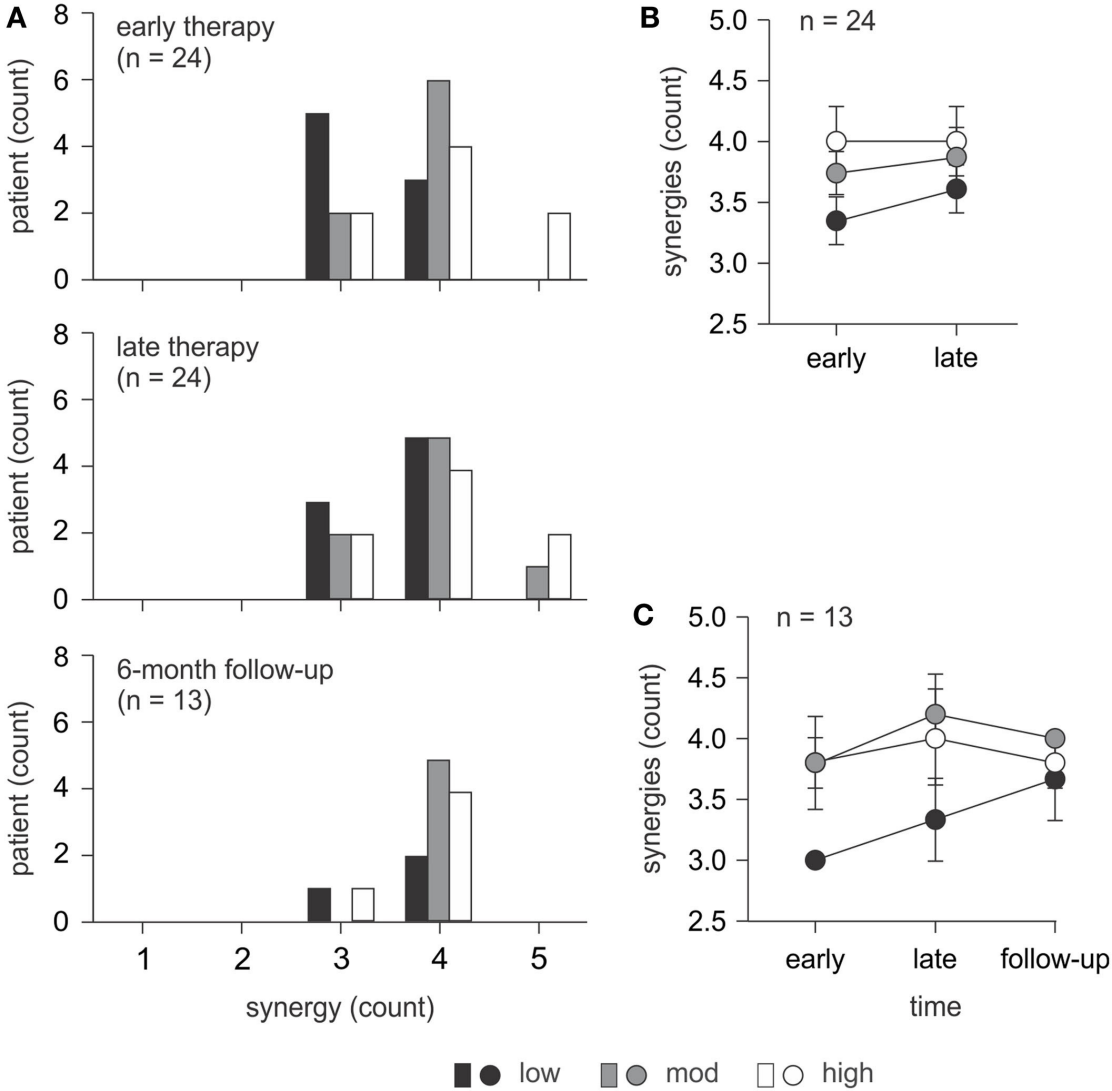
Number of synergies required to define Wii-baseball swing. **(A)** Comparison of the number of synergies used for patients (*n* = 24) with different levels of motor-function at early and late therapy and for a subset (*n* = 13) at 6-month follow-up. **(B)** The number of synergies for all patients (*n* = 24) at early and late therapy (mean ± SE). At early therapy, there was a significant difference between patients with low and high motor-function. There was also a trend toward an increase in the number of synergies from early to late therapy for patients with low and moderate motor-function. **(C)** The number of synergies for the subset of patients (*n* = 13; 3 low, 5 moderate, and 5 high motor-function) who completed 6-month follow-up assessments (mean ± SE). There was no significant change over time.

The number of muscle synergies required to define the Wii-baseball movement is presented in Figure 2A for each level of motor-function at each time point. At early therapy, most patients with low motor-function used three synergies, while most patients with moderate and high motor-function used four synergies to define the movement. However, two patients with high motor-function used five synergies. As can be seen in Figure 2B at early therapy, the number of synergies (mean ± SE) for patients with low motor-function (3.38 ± 0.18) was significantly less than for patients with high motor-function (4.00 ± 0.27) (*p* = 0.036). At early therapy, Fisher’s exact test showed no relationship between the number of muscle synergies and the level of motor-function (*p* = 0.217), although linear-by-linear association indicated a trend (*p* = 0.045).

#### Changes in the Number of Synergies over Time

At late therapy, an increase in the number of synergies was evident for patients with low and moderate motor-function, albeit not statistically significant (Figure 2B). The number of synergies (mean ± SE) increased from 3.38 ± 0.18 to 3.63 ± 0.18 (*p* = 0.317) for patients with low motor-function and from 3.75 ± 0.16 to 3.88 ± 0.23 (*p* = 0.564) for patients with moderate motor-function from early to late therapy. There was no change for patients with high motor-function. For the subset of patients with 6-month follow-up data, the number of synergies over time is illustrated in Figure 2C. There were no significant changes over time for this subset of patients.

### Consistency of Synergy Timing Profiles within Groups

**Figure 3:**
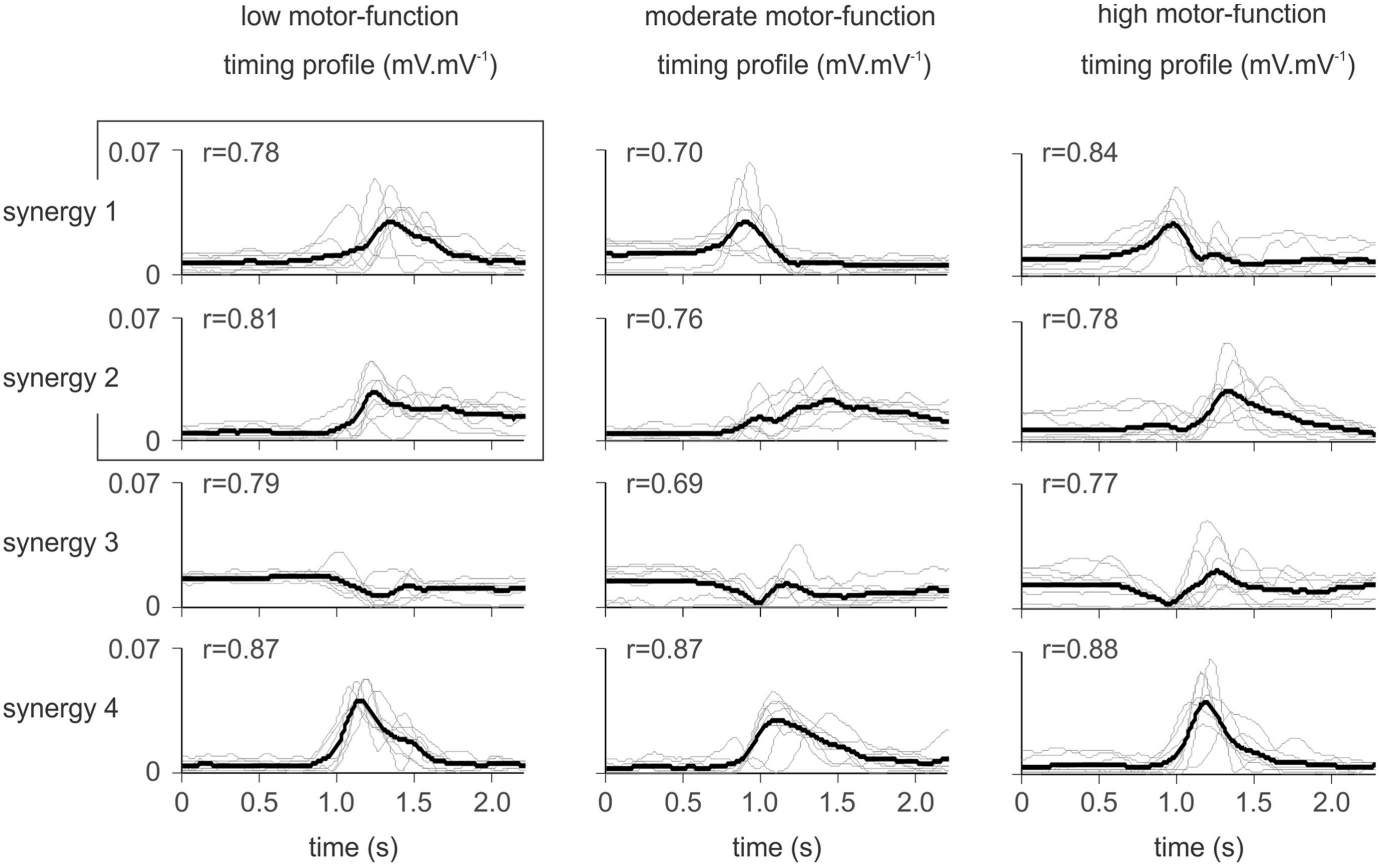
Synergy activation timing for patients according to the level of residual voluntary motor-function. Thin gray lines illustrate the synergy timing profile for each patient (mean of 10 trials), overlaid by the within-group mean (black line). Similar synergies are overlaid based on the scalar product between synergies. The *r*-value shown for each synergy indicates the group-averaged scalar product. For patients with low motor-function, the scalar product between mean synergy 1 and 2 indicated similarity (*r* = 0.71). These two synergies were assumed to be one (as indicated by the overlaid box). Thus, patients with low motor-function used three distinct synergies to define the movement, while patients with moderate and high motor-function required four synergies.

The synergy timing profiles (Figure 3) were similar for patients in each level of motor-function. The timing profile of muscle synergies in each group was matched based on the scalar product (*r*-value) between pairs of synergies from different patients in each group. The within-group mean *r*-value is shown for each synergy in Figure 3. Four distinct synergies demonstrated the profile of muscle synergies for patients with high and moderate motor-function. For patients with low motor-function, the between-synergy scalar product for synergy 1 and 2 (r = 0.71) showed high similarity, suggesting a single synergy. Thus, three distinct synergies defined the movement in patients with low motor-function.

### Variability (VAF) of Muscle Synergies across Groups

**Figure 4:**
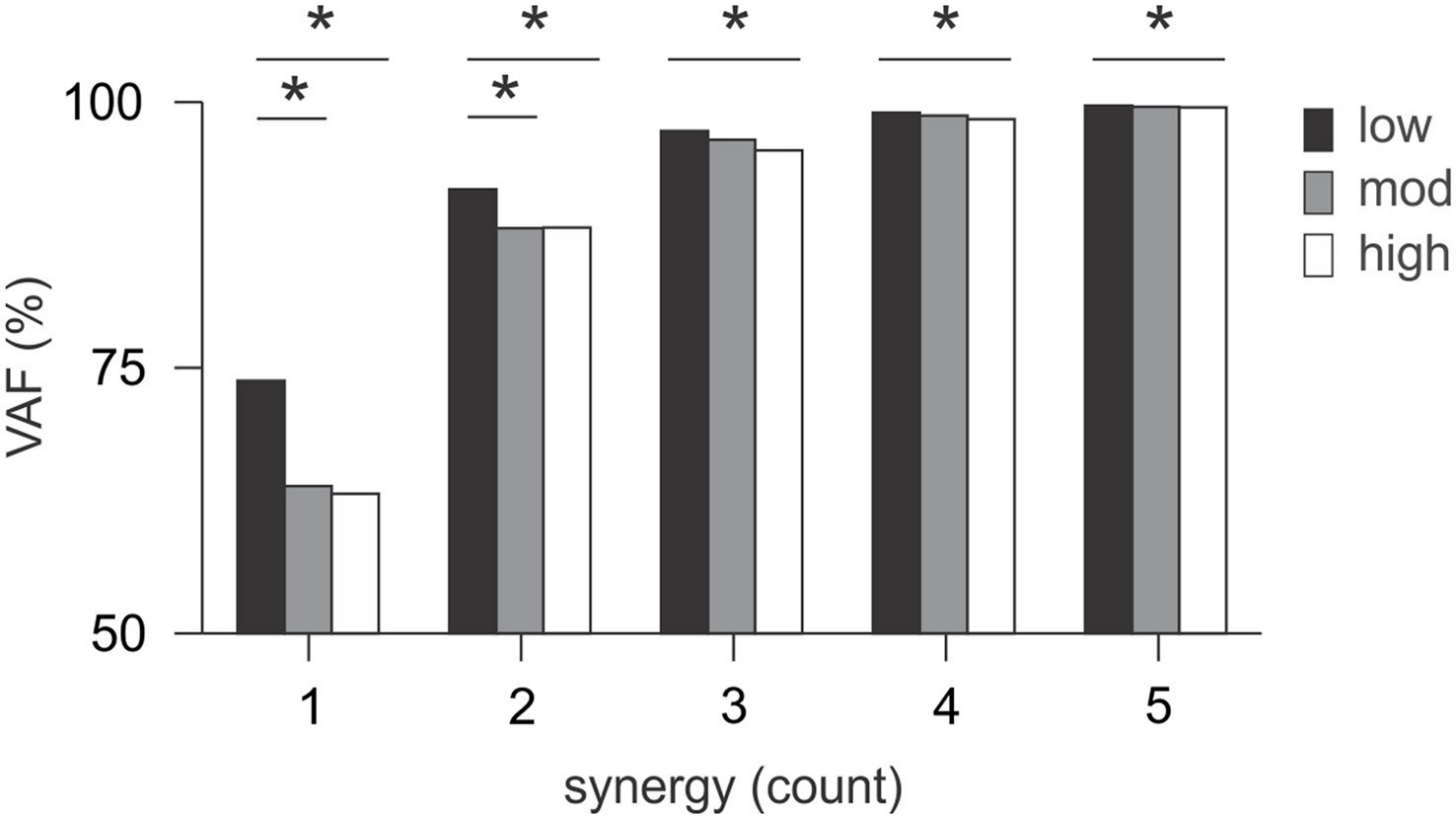
*Variability accounted for* (VAF) in muscle synergies. VAF changed little over time; the mean VAF was measured for any given number of synergies and compared between patients for the three levels of motor-function. For each number of synergies, patients with low motor-function had higher VAF compared to the other two groups (*p* < 0.05). Lower VAF for patients with moderate and high motor-function indicated that the analysis is less able to account for variability of muscle activation because of more movement complexity.

*Variability accounted for* increased with a higher number of synergies (Figure 4). A mixed-effect model revealed changes in VAF according to the level of motor-function over time for any given number of muscle synergies (range 1–5) for each patient. The level of motor-function, but not the time point, had an effect on the VAF (for any given number of synergies *p* < 0.05); although the VAF appears similar between groups in Figure 4, the variability within each group was large.

### Muscle Synergy Clusters

**Figure 5:**
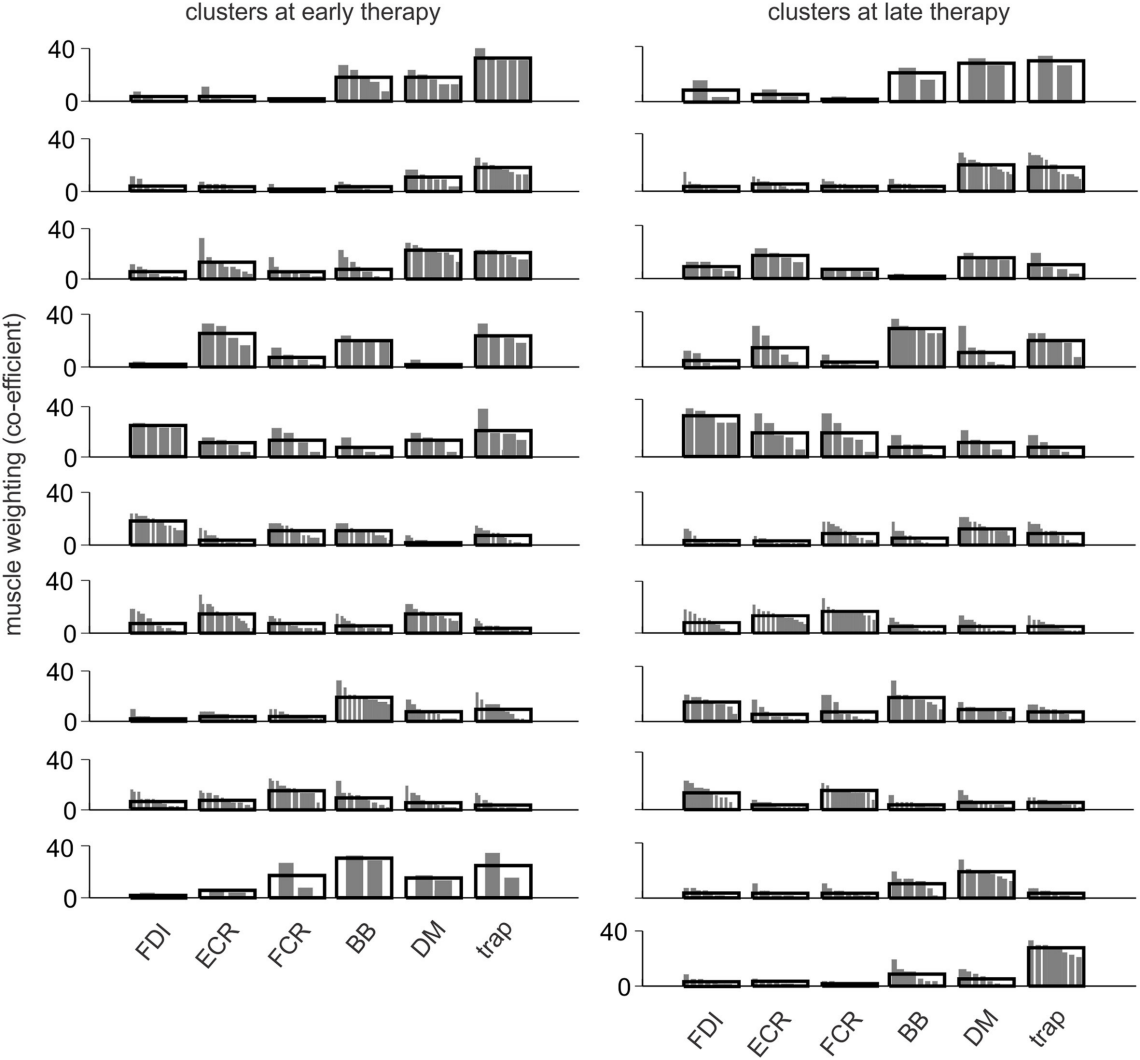
Composition of muscle synergies at early and late therapy. Synergy muscle weightings at early and late therapy were categorized into 10 and 11 clusters, respectively. For each cluster, the distribution of muscle weightings from different synergies is shown, overlaid by the group mean. The synergy clusters changed from early to late therapy except for the first four clusters.

**Figure 6:**
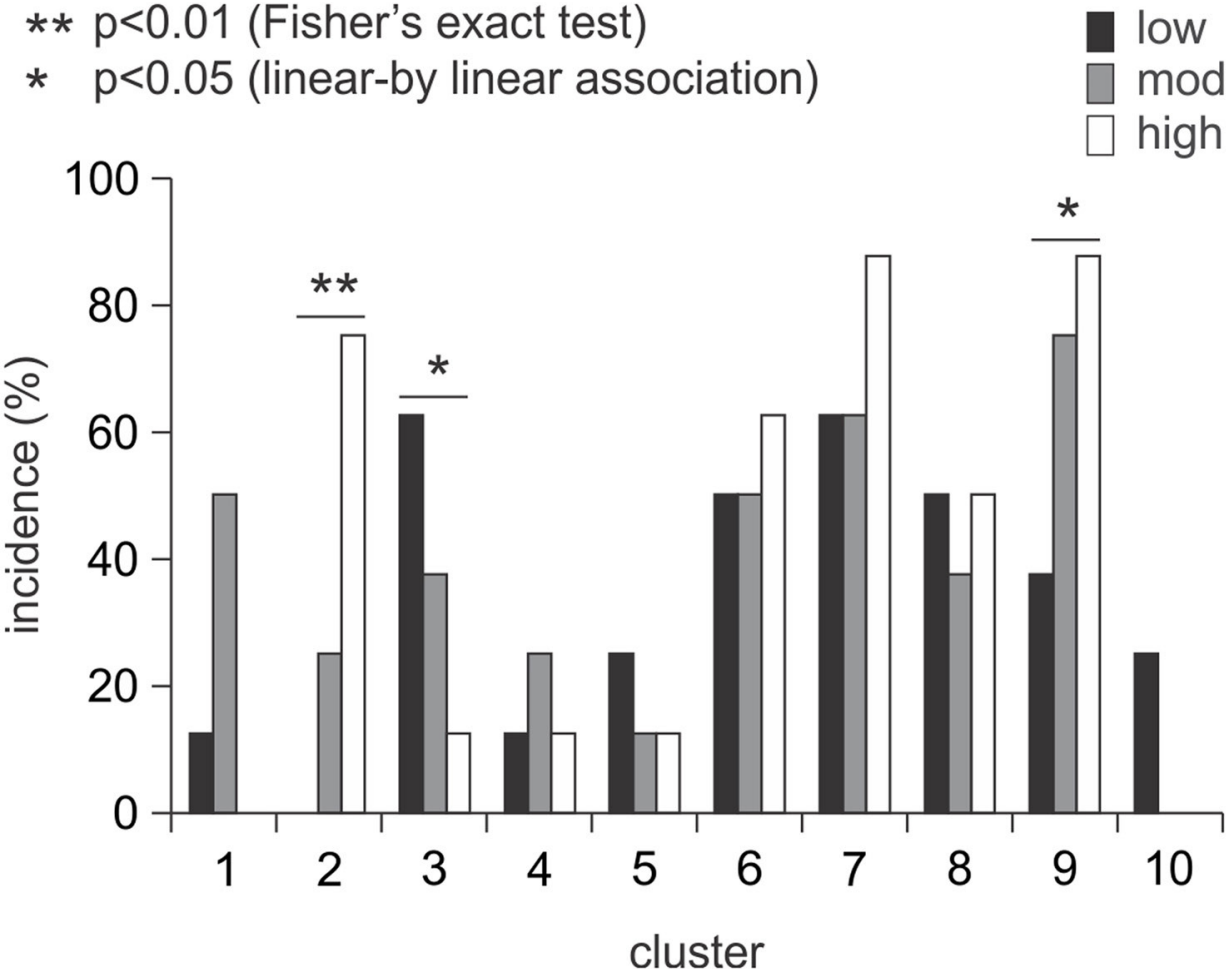
Incidence of muscle synergy clusters across groups at early therapy. The incidence of muscle synergies did not differ with the level of motor-function except for cluster 2 (*p* < 0.01). There was a trend between the incidence of muscle synergies and the level of motor-function in clusters 3 and 9 (*p* < 0.05).

At early- and late-therapy, the muscle weightings of each synergy from all patients were pooled and then categorized into 10 and 11 clusters, respectively. Thus, all the synergy structures from all patients can be summarized into 10 or 11 distinct synergies (Figure 5). At early therapy, there was no significant difference in the incidence of muscle synergies from different levels of patient motor-function in each cluster based on Fisher’s exact test, except for cluster 2 (Figure 6). However, a trend was observed in the incidence of cluster 3 and 9 using linear-by-linear association (see Figure 6).

### Wii-baseball Game Performance

The number of balls hit by patients was averaged for each recording session. The number of Wii-baseball hits increased (*p* < 0.001) from early therapy (4.42 ± 0.63) to late therapy (7.37 ± 0.40) and was sustained at 6-month follow-up (6.33 ± 0.65, *p* = 0.106). There was no difference in the duration of the Wii-baseball swing between groups (low, 1.30 ± 0.51 s; moderate, 1.37 ± 0.24 s; high, 0.75 ± 0.15 s; *p* = 0.379), i.e., there was no effect of motor-function level.

### Clinical Motor-Function Assessments

The clinical motor-function measures showed significant improvements from pre- to post-therapy. WMFT task times for the pooled data reduced (improved) from 38.1 ± 7.8 to 33.6 ± 7.2 s (*p* = 0.004), FMA scores increased from 46.6 ± 3.6 to 48.9 ± 3.6 (*p* = 0.001), and MALQOM scores of 60.1 ± 8.7 increased to 91.3 ± 8.1 (*p* < 0.001). All improvements were sustained at 6-month follow-up so that changes from post-therapy to the follow-up assessments were not significant (WMFT, *p* = 0.917; FMA, *p* = 0.107; MALQOM, *p* = 0.454).

## Discussion

In this longitudinal study, we identified and quantified muscle synergies *during* formal therapy sessions for patients with chronic stroke and different levels of motor-function at early and late therapy, and for a subset of patients at 6-month follow-up. As far as we can ascertain, this is the first study to investigate changes in the coordinated activation of muscles in chronic stroke during rehabilitation activities, rather than during unrelated clinical assessment tasks or restricted experimental tasks. The novel aspects of this study include a broad spectrum of poststroke residual voluntary motor-function; and the nature of the complex movement that was largely unconstrained, i.e., the start and end points were not experimentally predetermined. Therapist-guided quality of movement was the primary objective of the task during which EMG was recorded, and not the recording *per se* or game performance. Despite this, we identified differences in the number of muscle synergies used by patients as a function of the level of motor deficit. Thus, the profile of coordinated muscle activation varied by the level of residual motor-function in chronic stroke.

There is abundant evidence in the literature that motor ability is stable in the poststroke chronic period (52–54), even in the presence of some therapy protocols (55). It is also clear that targeted therapy can improve motor ability in contrast to control groups [(56, 57), see Ref. (58)]. The control groups receiving usual care in these studies provide further evidence of the stability of motor performance in chronic stroke for patients not receiving therapy or receiving usual care. In our setting, stable motor performance was established using pre-baseline to baseline testing in a randomized controlled study comparing WMT and modified Constraint-induced Movement Therapy (38).

The statistical outcomes in this study underestimate the level of information provided by this complex series of analyses and reflect the absence of a consistent pattern of change for between-patient EMG as identified in Hasam-Shariati et al. (Paper 1). The number of synergies used during Wii-baseball increased (although not significantly) with therapy for patients with low and moderate motor-function. At early therapy, there was a trend between the number of synergies and the level of motor-function that suggests different patterns of coordinated muscle activation between motor-function groups. The VAF of muscle synergies increased with a higher number of synergies, since the muscle activation can be defined more accurately with more synergies (i.e., smaller error) (17). VAF changed significantly with the level of motor-function but not over time within a level. Clustering the synergies from all patients showed that the incidence of three clusters has an association with the level of motor-function. Cluster analyses provide a means of demonstrating changes in the muscle weighting of some synergies between control and stroke groups (51). This suggests the distribution of muscle weightings within synergies in the present study changed as a consequence of therapy, as most synergy clusters changed from early to late therapy.

The similarity of muscle synergies has been investigated differently across studies. For example, the similarity of muscle weightings was used to demonstrate patients with different levels of motor ability used the same muscles during an isometric force generation task (51); whereas the timing profile was used to reveal different numbers of muscle synergies according to the level of motor ability during gait cycle (17). Our results reflect those of Clark et al. (17), in that the similarity of synergy timing profiles was used to distinguish the difference in the number of muscle synergies between groups: three distinct synergies defined the movement for patients with low motor-function, while four synergies were required for patients with moderate and high motor-function.

The coordination necessary to define a complex movement was associated with the level of residual voluntary motor-function but not the duration of the swing, with differences across time points not as evident as those shown in EMG analysis (42). This was confirmed by video recordings showing less complexity and more muscle co-activation for patients with low motor-function. Yet despite significant improvements in clinical assessments and Wii-baseball game performance, there was no difference in the number of muscle synergies over time. However, the change in the structure of muscle weightings from the cluster analysis at early and late therapy indicates that muscle recruitment changed between time points and that there was more diversity in muscle synergies after therapy.

Typically, muscle synergies for stroke patients are derived from stereotypical (1, 17) or experimentally constrained (30, 51) tasks. However, in this study, the movement was largely unconstrained. Although this may have reduced the sensitivity of the analysis, it is a better reflection of task-related real-world use of the upper limb after stroke. This approach also provides a more direct assessment of the neurophysiological changes induced by therapy (59). Stroke patients with different levels of motor-function use different strategies to resolve the same problem (task) (60). As highlighted by Hesam-Shariati et al. (42), the muscle characteristics for each patient differ depending on various neuromuscular limitations including weakness, hypertonicity, and spasticity. Such differences alter the goals of therapy (38) and result in more deliberate movement patterns than are seen in healthy control subjects (39).

The movement analyzed in this study was performed as part of a structured therapy program (61) with no attempt at standardization as would occur under experimental conditions (7, 51). Due to the range of motor impairment of the patients involved in this study, there were no standardized requirements for specific joint involvement or movement. The aim of this movement during therapy was to increase movement excursion (range of motion), velocity, strength, and control based on the generalized movement parameters of a baseball swing by a healthy subject. Although little attention was paid to the rules of the game, those for Wii-baseball provided some consistency in the patient striking response, in that the ball must be pitched (by the device) within a relatively small area (62). Thus, the onset of movement was determined through the delivery of the ball by the device. When the patient mistimed the movement and did not hit the ball but completed a swing, this movement was included in the analysis. While the start point of the movement was in an unrestricted task-dependant spatiotemporal framework, the end point, duration, speed, and direction were unconstrained *a priori* (38, 40).

The muscles contributing to a synergy varied from patient to patient and between patients within each level of motor-function. Synergy analysis provides a means of examining changes in motor coordination after stroke independent of the movement strategy of each patient (63). Our previous paper (42) focused on the dominant muscle activated during each activity. Here, synergy analyses provide a means of understanding how the brain coordinates neuromuscular control of movement (64) that can be used to build a dynamic model of the poststroke rehabilitation process.

Clearly, more than six muscles are necessary to produce the movement studied, even poststroke. We were limited in the number of channels available by the recording system and have previously reported EMG of tibialis anterior (59). The upper body muscles in this study were selected for two main reasons. First, they included a distribution along the neuromuscular axis of the more-affected side. Second, this recording montage limits the potential for EMG cross talk (65) while still reflecting the major muscle groups involved in the movement across the patient cohort (62). We incorporated EMG from the trapezius muscle in this analysis as a surrogate marker for trunk rotation where biceps and deltoid activation were insufficient to generate sufficient swing movements in Wii-baseball. EMG data from FDI were included to reflect the use of the hand during therapy because this muscle is readily accessible during therapy and was taken as a surrogate marker of intrinsic hand muscle activity. FDI activation was task dependant during Wii-baseball. EMG from triceps brachii was not recorded due to technical limitations including its very low level of activation compared to BB (66) and problems with loose skin in older patients which when combined with gravity acted to pull the sensor away from the muscle, rendering such recordings unreliable.

Synergy analysis addresses coordinated muscle activation (between muscles) rather than activation within each muscle. It is impossible after stroke to assume any similarity of underlying physiology and anatomy or to individually record the activity of each motor unit contributing to compound muscle activity. Any recording of EMG or method of EMG analysis will provide a biased estimate of activity (67, 68). The number of simultaneous recordings will not reduce the bias; in our experience, it increases the potential for cross talk and phase cancelation. Given the variability of impairment and ability after stroke both in the neuromusculature and factors impinging on the neuromusculature (e.g., somatosensation), in addition to the trial to trial variability for any given patient, it would be extremely difficult to estimate the ideal number of channels necessary for error-free synergy analyses.

### Clinical Implications

This study addresses the paucity of neurophysiological studies after stroke and as a consequence of therapy. This longitudinal investigation of changes in muscle synergies with therapy in chronic stroke across patients with different levels of motor-function provides initial insights into some of the neurophysiological mechanisms underpinning a therapy that is the equivalent of current best practice poststroke, namely, Constraint-induced Movement Therapy (38). Although there were few changes in the number of synergies, the altered structure of muscle synergies suggest that the coordination of muscle activation did improve and that this change was reflected in improved clinical assessment data (28) [presented in detail in Hesam-Shariati et al. (42)]. In particular, the significant improvements in MALQOM scores reflect greater independence in activities of everyday living (38).

This study demonstrates that the number of synergies, synergy timing profiles, distribution of muscle weightings, and VAF for muscle synergies differ according to the level of motor-function; particularly for patients with low motor-function at early therapy. These differences provide more detailed information about the neurophysiological functioning after stroke and how this changes with therapy. We hypothesize that altered muscle synergy structure reflects changes in brain connectivity, but this requires specific investigations of brain imaging or brain stimulation (69, 70). Nevertheless, the structure of muscle synergies can be used as an approach to classify stroke patients and to inform rehabilitation methods. However, muscle synergy analyses are insufficient on their own to fully understand neurophysiological changes with therapy after stroke and these analyses further emphasize the absence of any one tool to adequately quantify and explain the changes after stroke or with rehabilitation.

### Study Limitations

The primary focus of WMT is on the quality of movement, and increasing independent use of the more-affected upper limb in everyday tasks (38, 39). For this reason, therapy instructions are not those that would be used with healthy control subjects. For example, the different phases of the movement are emphasized differently depending on the level of motor impairment and may be practiced individually before being combined during the game performance using the principles of shaping (71), much like a sporting drill. Although the absence of healthy control subjects is a limitation of this study, the different movement patterns observed during game play (39) may limit the usefulness of such comparisons.

The sample size in this study is small within each level of motor-function. However, the total number of patients compares well with previous stroke studies investigating muscle synergies (18, 23, 30, 51). According to clinical assessment scores, this cohort included a wide range of residual voluntary motor capacity, particularly those with low motor-function who are rarely recruited in poststroke therapy and neurophysiology studies. This approach reduces the potential for statistically significant outcomes when data are pooled (59) but provides data that can be more readily generalized to the stroke population, although this study in chronic stroke cannot be generalized to the acute and subacute phase.

## Conclusion

Motor control differs for patients with different levels of residual voluntary motor-function when performing the same movement. Despite this, muscle synergies can be identified and monitored during therapy to understand changes in motor control of a largely unconstrained complex movement. A higher number of muscle synergies reflects greater movement complexity and task-related phasic muscle activation. This result is evidence for less complexity and more co-activation in the patterns of muscle activation for patients with low motor-function. The increased number of synergies by late therapy suggests improved motor control with more distinct phases of movement for patients with low motor-function. The change in the muscle synergy clusters by late therapy and different patterns of recovery indicate that the recruitment and activation of muscles change during therapy.

## Ethics Statement

This study was carried out in accordance with the recommendations of St. Vincent’s Hospital Human Research Ethics Committee with written informed consent from all patients. All patients gave written informed consent in accordance with the Declaration of Helsinki. The protocol was approved by the St. Vincent’s Hospital Human Research Ethics Committee, Sydney.

## Author Contributions

PMcN conceived, designed, and supervised the study and manuscript preparation. NH-S assisted with data collection, developed multiple code scripts, analyzed data, and drafted the manuscript, TT implemented data collection, AT-B implemented therapy, and CS undertook clinical assessments. All authors contributed to manuscript revision.

## Conflict of Interest Statement

The authors declare that the research was conducted in the absence of any commercial or financial relationships that could be construed as a potential conflict of interest.

## References

[1] Cheung VC Piron L Agostini M Silvoni S Turolla A Bizzi E. Stability of muscle synergies for voluntary actions after cortical stroke in humans. Proc Natl Acad Sci U S A (2009) 106(46):19563–8. 10.1073/pnas.0910114106 19880747 PMC2780765

[2] d’Avella A Lacquaniti F. Control of reaching movements by muscle synergy combinations. Front Comput Neurosci (2013) 7:42. 10.3389/fncom.2013.00042 23626534 PMC3630368

[3] Bizzi E Cheung V d’Avella A Saltiel P Tresch M. Combining modules for movement. Brain Res Rev (2008) 57(1):125–33. 10.1016/j.brainresrev.2007.08.004 18029291 PMC4295773

[4] d’Avella A Saltiel P Bizzi E Combinations of muscle synergies in the construction of a natural motor behavior. Nat Neurosci (2003) 6(3):300–8. 10.1038/nn1010 12563264

[5] Lee WA. Neuromotor synergies as a basis for coordinated intentional action. J Mot Behav (1984) 16(2):135–70. 10.1080/00222895.1984.10735316 14713663

[6] Tresch MC Saltiel P d’Avella A Bizzi E. Coordination and localization in spinal motor systems. Brain Res Rev (2002) 40(1):66–79. 10.1016/S0165-0173(02)00189-3 12589907

[7] Coscia M Cheung VC Tropea P Koenig A Monaco V Bennis C The effect of arm weight support on upper limb muscle synergies during reaching movements. Stroke (2014) 13(14):32–4. 10.1186/1743-0003-11-22 24594139 PMC3996016

[8] Safavynia SA Torres-Oviedo G Ting LH. Muscle synergies: implications for clinical evaluation and rehabilitation of movement. Top Spinal Cord Inj Rehabil (2011) 17(1):16–24. 10.1310/sci1701-16 21796239 PMC3143193

[9] Cheung VC d’Avella A Tresch MC Bizzi E. Central and sensory contributions to the activation and organization of muscle synergies during natural motor behaviors. J Neurosci (2005) 25(27):6419–34. 10.1523/JNEUROSCI.4904-04.2005 16000633 PMC6725265

[10] Tresch MC Saltiel P Bizzi E. The construction of movement by the spinal cord. Nat Neurosci (1999) 2(2):162–7. 10.1038/5721 10195201

[11] Kargo WJ Nitz DA. Early skill learning is expressed through selection and tuning of cortically represented muscle synergies. J Neurosci (2003) 23(35):11255–69. 14657185 10.1523/JNEUROSCI.23-35-11255.2003 PMC6741030

[12] Chvatal SA Macpherson JM Torres-Oviedo G Ting LH. Absence of postural muscle synergies for balance after spinal cord transection. J Neurophysiol (2013) 110(6):1301–10. 10.1152/jn.00038.2013 23803327 PMC3763149

[13] Ting LH Macpherson JM. A limited set of muscle synergies for force control during a postural task. J Neurophysiol (2005) 93(1):609–13. 10.1152/jn.00681.2004 15342720

[14] Torres-Oviedo G Macpherson JM Ting LH. Muscle synergy organization is robust across a variety of postural perturbations. J Neurophysiol (2006) 96(3):1530–46. 10.1152/jn.00810.2005 16775203

[15] Overduin SA d’Avella A Roh J Bizzi E. Modulation of muscle synergy recruitment in primate grasping. J Neurosci (2008) 28(4):880–92. 10.1523/JNEUROSCI.2869-07.2008 18216196 PMC6671000

[16] Chvatal SA Ting LH. Common muscle synergies for balance and walking. Front Comput Neurosci (2013) 7:48. 10.3389/fncom.2013.00048 23653605 PMC3641709

[17] Clark DJ Ting LH Zajac FE Neptune RR Kautz SA. Merging of healthy motor modules predicts reduced locomotor performance and muscle coordination complexity post-stroke. J Neurophysiol (2010) 103(2):844–57. 10.1152/jn.00825.2009 20007501 PMC2822696

[18] Coscia M Monaco V Martelloni C Rossi B Chisari C Micera S. Muscle synergies and spinal maps are sensitive to the asymmetry induced by a unilateral stroke. J Neuroeng Rehabil (2015) 12(1):39. 10.1186/s12984-015-0031-7 25928264 PMC4411739

[19] Chvatal SA Torres-Oviedo G Safavynia SA Ting LH. Common muscle synergies for control of center of mass and force in nonstepping and stepping postural behaviors. J Neurophysiol (2011) 106(2):999–1015. 10.1152/jn.00549.2010 21653725 PMC3154805

[20] Torres-Oviedo G Ting LH. Muscle synergies characterizing human postural responses. J Neurophysiol (2007) 98(4):2144–56. 10.1152/jn.01360.2006 17652413

[21] Ajiboye AB Weir R. Muscle synergies as a predictive framework for the EMG patterns of new hand postures. J Neural Eng (2009) 6(3):036004. 10.1088/1741-2560/6/3/036004 19436081 PMC3151158

[22] Zariffa J Steeves J Pai DK. Changes in hand muscle synergies in subjects with spinal cord injury: characterization and functional implications. J Spinal Cord Med (2012) 35(5):310–8. 10.1179/2045772312Y.0000000037 23031168 PMC3459560

[23] Cheung VC Turolla A Agostini M Silvoni S Bennis C Kasi P Muscle synergy patterns as physiological markers of motor cortical damage. Proc Natl Acad Sci U S A (2012) 109(36):14652–6. 10.1073/pnas.1212056109 22908288 PMC3437897

[24] Borzelli D Berger DJ Pai D D’avella A. Effort minimization and synergistic muscle recruitment for three-dimensional force generation. Front Comput Neurosci (2013) 7:186. 10.3389/fncom.2013.00186 24391581 PMC3868911

[25] Roh J Rymer WZ Beer RF. Robustness of muscle synergies underlying three-dimensional force generation at the hand in healthy humans. J Neurophysiol (2012) 107(8):2123–42. 10.1152/jn.00173.2011 22279190 PMC3331600

[26] Gizzi L Nielsen JF Felici F Ivanenko YP Farina D Impulses of activation but not motor modules are preserved in the locomotion of subacute stroke patients. J Neurophysiol (2011) 106(1):202–10. 10.1152/jn.00727.2010 21511705

[27] Hidler JM Carroll M Federovich EH Strength and coordination in the paretic leg of individuals following acute stroke. IEEE Trans Neural Syst Rehabil Eng (2007) 15(4):526–34. 10.1109/TNSRE.2007.907689 18198710

[28] Tropea P Monaco V Coscia M Posteraro F Micera S. Effects of early and intensive neuro-rehabilitative treatment on muscle synergies in acute post-stroke patients: a pilot study. J Neuroeng Rehabil (2013) 10(1):103. 10.1186/1743-0003-10-103 24093623 PMC3850948

[29] Dipietro L Krebs HI Fasoli SE Volpe BT Stein J Bever C Changing motor synergies in chronic stroke. J Neurophysiol (2007) 98(2):757–68. 10.1152/jn.01295.2006 17553941

[30] Li S Zhuang C Zhang X Niu CM Xie Q Lan N Analysis of muscle synergy for evaluation of task-specific performance in stroke patients. Paper Presented at the Engineering in Medicine and Biology Society (EMBC), 2016 IEEE 38th Annual International Conference Orlando, FL (2016). 10.1109/EMBC.2016.7591041 28268653

[31] Roh J Rymer WZ Perreault EJ Yoo SB Beer RF. Alterations in upper limb muscle synergy structure in chronic stroke survivors. J Neurophysiol (2013) 109(3):768–81. 10.1152/jn.00670.2012 23155178 PMC3567389

[32] Werring DJ Toosy AT Clark CA Parker GJ Barker GJ Miller DH Diffusion tensor imaging can detect and quantify corticospinal tract degeneration after stroke. J Neurol Neurosurg Psychiatry (2000) 69(2):269–72. 10.1136/jnnp.69.2.269 10896709 PMC1737065

[33] Ramsay JW Barrance PJ Buchanan TS Higginson JS. Paretic muscle atrophy and non-contractile tissue content in individual muscles of the post-stroke lower extremity. J Biomech (2011) 44(16):2741–6. 10.1016/j.jbiomech.2011.09.001 21945568 PMC3208767

[34] Kautz S Brown D. Relationships between timing of muscle excitation and impaired motor performance during cyclical lower extremity movement in post-stroke hemiplegia. Brain (1998) 121(3):515–26. 10.1093/brain/121.3.515 9549527

[35] Tresch MC Cheung VC d’Avella A. Matrix factorization algorithms for the identification of muscle synergies: evaluation on simulated and experimental data sets. J Neurophysiol (2006) 95(4):2199–212. 10.1152/jn.00222.2005 16394079

[36] Chiovetto E Berret B Delis I Panzeri S Pozzo T. Investigating reduction of dimensionality during single-joint elbow movements: a case study on muscle synergies. Front Comput Neurosci (2013) 7:11. 10.3389/fncom.2013.00011 23450667 PMC3584318

[37] Frère J Hug F. Between-subject variability of muscle synergies during a complex motor skill. Front Comput Neurosci (2012) 6:99. 10.3389/fncom.2012.00099 23293599 PMC3531715

[38] McNulty PA Thompson-Butel AG Faux SG Lin G Katrak PH Harris LR The efficacy of Wii-based Movement Therapy for upper limb rehabilitation in the chronic poststroke period: a randomized controlled trial. Int J Stroke (2015) 10(8):1253–60. 10.1111/ijs.12594 26332338

[39] Mouawad MR Doust CG Max MD McNulty PA. Wii-based Movement Therapy to promote improved upper extremity function post-stroke: a pilot study. J Rehabil Med (2011) 43(6):527–33. 10.2340/16501977-0816 21533334

[40] Trinh T Scheuer SE Thompson-Butel AG Shiner CT McNulty PA Cardiovascular fitness is improved post-stroke with upper-limb Wii-based Movement Therapy but not dose-matched constraint therapy. Top Stroke Rehabil (2016) 23(3):208–16. 10.1080/10749357.2016.1138672 26907502

[41] Casadio M Tamagnone I Summa S Sanguineti V. Neuromotor recovery from stroke: computational models at central, functional, and muscle synergy level. Front Comput Neurosci (2013) 7:97. 10.3389/fncom.2013.00097 23986688 PMC3749429

[42] Hesam-Shariati N Trinh T Thompson-Butel AG Shiner CT McNulty PA A longitudinal electromyography study of complex movements in poststroke therapy. 1: heterogenous changes despite consistent improvements in clinical assessments. Front Neurol (2017). 10.3389/fneur.2017.00340 PMC5532386 28804474

[43] Thompson-Butel AG Lin GG Shiner CT McNulty PA. Two common tests of dexterity can stratify upper limb motor function after stroke. Neurorehabil Neural Repair (2014) 28(8):788–96. 10.1177/1545968314523678 24627336

[44] Shiner CT Pierce KD Thompson-Butel AG Trinh T Schofield PR McNulty PA. BDNF genotype interacts with motor function to influence rehabilitation responsiveness poststroke. Front Neurol (2016) 7:69. 10.3389/fneur.2016.00069 27242654 PMC4868962

[45] Wolf SL Lecraw DE Barton LA Jann BB Forced use of hemiplegic upper extremities to reverse the effect of learned nonuse among chronic stroke and head-injured patients. Exp Neurol (1989) 104(2):125–32. 10.1016/S0014-4886(89)80005-6 2707361

[46] Fugl-Meyer AR Jääskö L Leyman I Olsson S Steglind S The post-stroke hemiplegic patient. 1. a method for evaluation of physical performance. Scand J Rehabil Med (1974) 7(1):13–31. 1135616

[47] Uswatte G Taub E Morris D Vignolo M McCulloch K Reliability and validity of the upper-extremity Motor Activity Log-14 for measuring real-world arm use. Stroke (2005) 36(11):2493–6. 16224078 10.1161/01.STR.0000185928.90848.2e

[48] Lee DD Seung HS. Learning the parts of objects by non-negative matrix factorization. Nature (1999) 401(6755):788–91. 10.1038/44565 10548103

[49] Paatero P Tapper U Positive matrix factorization: a non-negative factor model with optimal utilization of error estimates of data values. Environmetrics (1994) 5(2):111–26. 10.1002/env.3170050203

[50] Berry MW Browne M Langville AN Pauca VP Plemmons RJ Algorithms and applications for approximate nonnegative matrix factorization. Comput Stat Data Anal (2007) 52(1):155–73. 10.1016/j.csda.2006.11.006

[51] Roh J Rymer WZ Beer RF. Evidence for altered upper extremity muscle synergies in chronic stroke survivors with mild and moderate impairment. Front Hum Neurosci (2015) 9:6. 10.3389/fnhum.2015.00006 25717296 PMC4324145

[52] Krakauer JW. Motor learning: its relevance to stroke recovery and neurorehabilitation. Curr Opin Neurol (2006) 19(1):84–90. 10.1097/01.wco.0000200544.29915.cc 16415682

[53] Mirbagheri MM Rymer WZ. Time-course of changes in arm impairment after stroke: variables predicting motor recovery over 12 months. Arch Phys Med Rehabil (2008) 89(8):1507–13. 10.1016/j.apmr.2008.02.017 18586221

[54] Stinear C. Prediction of recovery of motor function after stroke. Lancet Neurol (2010) 9(12):1228–32. 10.1016/S1474-4422(10)70247-7 21035399

[55] Mehrholz J Pohl M Platz T Kugler J Elsner B Electromechanical and robot‐assisted arm training for improving activities of daily living, arm function, and arm muscle strength after stroke. Cochrane Database Syst Rev (2015) (11):CD006876. 10.1002/14651858.CD006876.pub4 26559225 PMC6465047

[56] Page SJ Levine P Leonard A Szaflarski JP Kissela BM. Modified constraint-induced therapy in chronic stroke: results of a single-blinded randomized controlled trial. Phys Ther (2008) 88(3):333. 10.2522/ptj.20060029 18174447

[57] Wolf SL Winstein CJ Miller JP Taub E Uswatte G Morris D Effect of constraint-induced movement therapy on upper extremity function 3 to 9 months after stroke: the EXCITE randomized clinical trial. JAMA (2006) 296(17):2095–104. 10.1001/jama.296.17.2095 17077374

[58] Teasell RW Murie Fernandez M McIntyre A Mehta S. Rethinking the continuum of stroke rehabilitation. Arch Phys Med Rehabil (2014) 95(4):595–6. 10.1016/j.apmr.2013.11.014 24529594

[59] Trinh T Shiner CT Thompson-Butel AG McNulty PA Targeted upper-limb Wii-based Movement Therapy also improves lower-limb muscle activation and functional movement in chronic stroke. Disabil Rehabil (2016):1–11. 10.1080/09638288.2016.1213892 27718640

[60] Cirstea M Levin MF. Compensatory strategies for reaching in stroke. Brain (2000) 123(5):940–53. 10.1093/brain/123.5.940 10775539

[61] McNulty PA. Games for rehabilitation: Wii-based Movement Therapy improves poststroke movement ability. Games Health J (2012) 1(5):384–7. 10.1089/g4h.2012.0055 26192005

[62] Deutsch JE Brettler A Smith C Welsh J John R Guarrera-Bowlby P Nintendo Wii sports and Wii fit game analysis, validation, and application to stroke rehabilitation. Top Stroke Rehabil (2011) 18(6):701–19. 10.1310/tsr1806-701 22436308

[63] McMorland AJ Runnalls KD Byblow WD. A neuroanatomical framework for upper limb synergies after stroke. Front Hum Neurosci (2015) 9:82. 10.3389/fnhum.2015.00082 25762917 PMC4329797

[64] Ting LH McKay JL. Neuromechanics of muscle synergies for posture and movement. Curr Opin Neurobiol (2007) 17(6):622–8. 10.1016/j.conb.2008.01.002 18304801 PMC4350235

[65] Hug F. Can muscle coordination be precisely studied by surface electromyography? J Electromyogr Kinesiol (2011) 21(1):1–12. 10.1016/j.jelekin.2010.08.009 20869882

[66] Bowden JL Taylor JL McNulty PA. Voluntary activation is reduced in both the more-and less-affected upper limbs after unilateral stroke. Front Neurol (2014) 5:239. 10.3389/fneur.2014.00239 25477862 PMC4237055

[67] De Luca CJ Gilmore LD Kuznetsov M Roy SH. Filtering the surface EMG signal: movement artifact and baseline noise contamination. J Biomech (2010) 43(8):1573–9. 10.1016/j.jbiomech.2010.01.027 20206934

[68] Tibold R Fuglevand AJ Prediction of muscle activity during loaded movements of the upper limb. J Neuroeng Rehabil (2015) 12(1):6 10.1186/1743-0003-12-6 25592397 PMC4326445

[69] Bajaj S Butler AJ Drake D Dhamala M Brain effective connectivity during motor-imagery and execution following stroke and rehabilitation. Neuroimage Clin (2015) 8:572–82. 10.1016/j.nicl.2015.06.006 26236627 PMC4501560

[70] Rathee D Cecotti H Prasad G Estimation of effective fronto-parietal connectivity during motor imagery using partial granger causality analysis. Paper Presented at the Neural Networks (IJCNN), 2016 International Joint Conference, Vancouver (2016).

[71] Taub E Crago JE Burgio LD Groomes TE Cook EW DeLuca SC An operant approach to rehabilitation medicine: overcoming learned nonuse by shaping. J Exp Anal Behav (1994) 61(2):281–93. 10.1901/jeab.1994.61-281 8169577 PMC1334416

